# Hunterian School of Medicine

**Published:** 1852-11

**Authors:** 


Hunterian School of Medicine. *Winter Session.*—Principles and Practice of Medicine, Dr. Aldis. Principles and Practice of Surgery, Mr. Dampier. Anatomy and Physiology, Mr. Chippendale. Descriptive and Surgical Anatomy, Mr. Grant. Chemistry, Mr. Ashley. *Summer Session.*—Materia Medica, Dr. Smyth. Midwifery and Diseases of Women and Children, Dr. Gordon Bailey. Morbid Anatomy, Mr. H. Thompson. Practical Chemistry, Mr. Ashley. Medical Botany, Mr. C. P. Johnson. Forensic Medicine, Dr. R. Chambers.

